# Low-Potassium Fruits and Vegetables: Research Progress and Prospects

**DOI:** 10.3390/plants13141893

**Published:** 2024-07-09

**Authors:** Jiawei Cui, Yongxue Zhang, Hongmei Zhang, Haijun Jin, Lizhong He, Hong Wang, Panling Lu, Chen Miao, Jizhu Yu, Xiaotao Ding

**Affiliations:** Shanghai Key Laboratory of Protected Horticultural Technology, Horticulture Research Institute, Shanghai Academy of Agricultural Sciences, Shanghai 201403, China; cuijiawei@saas.sh.cn (J.C.); xuezylemon@foxmail.com (Y.Z.); zhanghongmei@saas.sh.cn (H.Z.); jinhaijun@saas.sh.cn (H.J.); hlznd02@163.com (L.H.); wanghong@saas.sh.cn (H.W.); lpl2245@163.com (P.L.); miaochen@saas.sh.cn (C.M.)

**Keywords:** chronic kidney disease (CKD), hyperkalemia, soilless culture, potassium utilization efficiency (KUE), tolerance to low-potassium (LK) stress

## Abstract

With the increasing number of patients with chronic kidney disease (CKD) and the improved recognition of nutritional therapy, research on low-potassium (LK) fruits and vegetables for CKD patients has gained global attention. Despite its already commercial availability primarily in Japan, public awareness remains limited, and cultivation methods lack a comprehensive strategy. This review offers an extensive examination of the developmental significance, current cultivation techniques, and existing limitations of functional LK fruits and vegetables with the objective of providing guidance and inspiration for their exploitation. Additionally, this review investigates various factors influencing K content, including varieties, temperature, light, exogenous substances, harvest time, and harvest parts, with a focus on optimizing production methods to enhance potassium utilization efficiency (KUE) and decrease the K content in plants. Finally, the review outlines the shortcomings and prospects of research on LK fruits and vegetables, emphasizing the importance of interdisciplinary research (in agriculture technology, medicine, and business) for patients with CKD and the future development of this field.

## 1. Introduction

Potassium ions (K^+^) are the most abundant cation in plants (non-halophytes) [1]. They are involved in a variety of plant functions, including osmoregulation and cell extension, stomatal movement, activation of enzymes, protein synthesis, photosynthesis, stress responses, phloem loading, and phloem transport and uptake [2,3]. Adequate K supply maintains the normal root morphology [4], enhances water and nutrient uptake [4,5], improves the photosynthetic efficiency [6], promotes the growth of plants [7], and meliorates the quality of fruits [8]. Although agriculture generally maintains high yield and high quality in fruits and vegetables by increasing the potassium content in plants [7,9], low-potassium (LK) fruits and vegetables for chronic kidney disease (CKD) patients have recently become a research hotspot [10,11].

CKD has become a significant global health concern, affecting an estimated 8–13% of the global population [12]. CKD is prevalent among the elderly, and with the increasing aging of society in certain countries, its prevalence is rising [13]. In CKD patients with impaired renal function, there is a decreased capacity to excrete K, leading to an increased risk of hyperkalemia [14]. Hyperkalemia causes symptoms such as a slow heart rate, numbness and soreness of limbs, and, in the worst cases, may lead to flaccid hemiplegia or cardiac arrest [15]. Consequently, CKD patients are advised to restrict high-K foods, with a recommended daily K intake below 3 g [16]. Fresh fruits and vegetables typically have a high K content. However, restricting their consumption can lead to deficiencies in essential vitamins and minerals [17], as well as nutrients rich in fiber and alkali, which can result in constipation and metabolic acidosis, both of which are significant risk factors for hyperkalemia [18]. Incorporating LK fruits and vegetables into CKD patient diets offers a solution to balancing nutritional needs while limiting K intake [19]. Therefore, investigations and applications of functional LK fruits and vegetables hold significant promise for individuals with CKD. Currently, there is no universally acknowledged K value for classifying fruits and vegetables as LK. In Japan, fruits and vegetables with a K content below 100 mg per 100 g of fresh weight (FW) are considered LK fruits and vegetables [20].

Investigations of LK fruits and vegetables for patients with CKD originated in Japan [21]. Various studies have reported research on LK fruits and vegetables, encompassing spinach [10], lettuce [22], strawberry [23], melon [24], onion [11] and tomato [25]. The Aizu-Wakamatsu Akisai Vegetable Plant Factory of Fujitsu has achieved a notable production of 2500 heads of LK lettuce daily, establishing itself as Japan’s largest plant factory for cultivating LK vegetables [20]. Studies on LK fruits and vegetables conducted in other countries such as China [26], South Korea [27], and Italy [19] have also been documented.

In this review, we present various existing methods for producing LK fruits and vegetables, outline the factors affecting the K content in plants, and suggest avenues for the further optimization of production methods.

## 2. Cultivation Methods of LK Fruits and Vegetables

Because the mineral elements in the nutrient solution of soilless cultivation are highly controllable, the production of LK fruits and vegetables primarily relies on soilless cultivation. This can be achieved by reducing the supply of K to the nutrient solution [26,28]. Production methods are categorized into two types based on nutrient solution management: LK supply via electrical conductance management (LKEC) and LK supply via nutrient quantitative management (LKQM) [28].

### 2.1. LKEC Method

The LKEC method refers to the reduction in the K supply while maintaining the EC of the nutrient solution during the cultivation of fruits and vegetables [28]. Conductivity managements are commonly used for fertilizer and water control in soilless cultivation. This involves establishing a target EC based on plant fertilizer demand. Fertilizers are added when the nutrient solution falls below the target EC value, whereas water can be added when it exceeds this value. The LKEC method operates on the principle of managing the nutrient solution EC.

The LKEC method encompasses three adjustment approaches to the nutrient solution formula. The first method involves reducing the application of K nitrate (KNO_3_) (the KNO_3_ reduction method). The second method maintains the levels of other mineral elements while only decreasing the K element (single-K element reduction method). For example, KNO_3_ in formula fertilizers is substituted with ammonium nitrate (NH_4_NO_3_). The third method maintains the nitrate ion (NO_3_^−^) content while substituting the K ions (K^+^) with sodium ions (Na^+^) or calcium ions (Ca^2+^) (cation substitution method). For example, KNO_3_ is replaced with sodium nitrate (NaNO_3_) or calcium nitrate (Ca(NO_3_)_2_) in formula fertilizers.

The first LKEC option is the KNO_3_ reduction method. K in the soilless cultivation nutrient solutions is commonly supplied as KNO_3_. Therefore, reducing the application of KNO_3_ could decrease the supply of K. Asaduzzaman [24] observed that melon plants in perlite culture, receiving only 50% of the required KNO_3_ during the 3rd and 4th weeks after transplantation, produced fruits with 53% less K content than the control group. The resulting LK melon was well received by patients with CKD owing to its appealing taste and the absence of a tingling sensation. Fuad Mondal [23] attempted to produce LK strawberry fruits by reducing the KNO_3_ fertilizer in the nutrient solution from anthesis to harvest. The ‘Toyonoka’ variety exhibited a 64% reduction in K content when cultivated in a nutrient solution containing KNO_3_ at 1/16 of the standard concentration.

The second LKEC option is the single-K element reduction method. Substituting NH_4_NO_3_ for KNO_3_ can reduce the K element while maintaining the nitrogen levels. This approach decreases the K content in vegetables. Liu [26] utilized NH_4_NO_3_ to replace a portion of KNO_3_ in the formula. As the K concentration in the nutrient solution decreased, the K content in the mustard, lettuce, and spinach exhibited a significant decline. The mustard, lettuce, and spinach treated with 1/8 K exhibited K contents 79.93%, 66.37%, and 60.33% lower than that of the normal K concentration, respectively. Moreover, the 1/8 K treatment presented higher levels of soluble solids and soluble sugar than the control group, while nitrogen, phosphorus, Ca and magnesium levels remained similar to the control group [26].

Last, there is the cation substitution method. Studies have indicated that Na^+^ and Ca^2+^ can potentially substitute for K^+^ in certain nonspecific physiological functions, such as osmoregulation [29,30]. This method of substituting K^+^ with Na^+^ or Ca^2+^ can mitigate the stress induced by LK levels in plants and concurrently reduce the K content. At present, the method of using Ca(NO_3_)_2_ or NaNO_3_ to replace part of the KNO_3_ in the nutrient solution has successfully produced kale [27], lettuce [31] and microgreens [19] and other vegetables with low potassium content, and these vegetable yields are no different from the control. Son et al. [27] demonstrated this by substituting KNO_3_ with Ca(NO_3_)_2_ 3 weeks after the kale planting, maintaining the same EC value. After 3 weeks of K deprivation treatment, the K content of kale demonstrated a 70% reduction in K content without any adverse effects on yield or visual quality. Additionally, an increase in the health-promoting component, glucosinolates, was observed. Renna et al. [19] successfully reduced the K content in seedling vegetables by using NaNO_3_ to replace either all or 75% of KNO_3_ in the nutrient solution. This resulted in seedling vegetables containing only 103–129 mg of K per 100 g of FW compared to the normal K supply of 225–250 mg K per 100 g of FW. The K content of the three Astericae seedlings (‘Molfetta’, ‘Italico a costa rossa’, and ‘Bionda da taglio’) decreased by approximately 50%, and the FW slightly decreased (17.5%). There were no significant differences in plant height, dry matter, or commercial value. Furthermore, there were no negative effects on the nutritional quality (total lipid, protein, total carbohydrate, fiber, ashes, and antioxidant activity).

Although all three methods can yield fruits and vegetables with LK content, the determination of the method should be based on the specific characteristics of the fruits and vegetables species. When employing the cation substitution method, consideration should be taken to the tolerance of fruits and vegetables to Na and Ca stress. Liu [26] compared the effects of the single-K element reduction method and the cation substitution method on the growth, mineral elements, and quality of three leafy vegetables (mustard, lettuce, and spinach). The results indicated that when the K supply level in the nutrient solution was below 1.19 mmol·L^−1^ for both methods, the K content in mustard and lettuce decreased by over 50%. The cation substitution method was more suitable for the LK production of mustard and lettuce, as it did not adversely affect yield. Conversely, the single-K element reduction method significantly reduced the yield of mustard and lettuce. Spinach could be sensitive to Na, and high Na levels inhibited the growth. Of both methods, the single K reduction method has little effect on the spinach yield, making it more suitable for spinach cultivation.

The timing of LK treatment could be crucial for implementing LKEC. Zhang et al. [22] demonstrated that initiating LK nutrient solution from the time of colonization led to reduced lettuce yield, rendering it ineffective for the LK lettuce production. However, Ogawa [31] has suggested that withholding K application during the latter half of the growth period for three types of leafy vegetables presented no difference in fresh and dry weights compared to the control group. This suggests that providing K normally during the early growth stage and then removing it during the late growth stage is preferred for LK production in fruits and vegetables [31]. K can be removed from the nutrient solution 1 or 2 weeks before harvesting for leafy vegetables [31], after flowering for solanaceous and cucurbitaceous plants [24], and at the bulb expansion stage for bulb vegetables [11].

### 2.2. LKQM Method

Researchers have employed the LKQM method to distribute the K requirements of plants across different stages of their growth [28]. A novel approach to nutrient management in hydroponics has emerged, in which fertilizer is regularly supplied to the cultivation system (quantitative management) regardless of the EC of the solution [20,32]. Despite fluctuations in nutrient levels with this method, researchers have suggested that the roots can efficiently absorb ions in large solution volumes without impeding plant growth [33]. This concept has been applied to the K supply for cultivating LK fruits and vegetables. Some researchers have successfully produced LK vegetables by using the LKQM method. In a LK cherry tomato cultivation experiment [25], the total quantity of K supply per plant during the cultivation was 7.2 g (1 K, set as control), 3.6 g (1/2 K), 1.8 g (1/4 K), 0.9 g (1/8 K) and 0.6 g (1/12 K), respectively, which means five quantitative treatments of K fertilizer were set in the experiment. Researchers allocated 25%, 50%, and 25% of the total designated K across three stages (before the first flower anthesis, after the first flower anthesis, and after the green maturity of the first truss) in respective treatments with the K amount evenly distributed for weekly application. When the total designed K was set at 1/4 K and 1/8 K, the average K content of fruits decreased from 151.8 mg·100 g^−1^ FW in the control to 107.6 mg·100 g^−1^ FW and 76.4 mg·100 g^−1^ FW, respectively. However, the fruit yield, sugar content, and acid content remained largely unchanged compared with the control. Okada et al. [11] investigated the amount of K absorbed at the onion bulb expansion stage under a sufficient amount of fertilizer. Then, they evenly distributed 1.5 times the amount of mineral elements absorbed during onion bulb expansion over the 15 weeks of the expansion stage, resulting in LK onions with a K content of 88.3 mg·100 g^−1^ FW, which was 41.1% lower than the value (150 mg·100 g^−1^ FW) reported in the Standard Tables of Food Composition in Japan (2015). Nevertheless, this had little impact on bulb yield.

The LKQM method appears to offer more advantages than the LKEC method. Xu et al. [28] conducted a comparison of their effects on lettuce growth and K content. They observed no significant differences in K content and plant growth between the LKEC and LKQM treatments. However, lettuce treated with LKQM demonstrated lower Na content than that treated with LKEC, and the overall fertilizer usage was reduced.

## 3. Optimization of the Cultivation Methods of LK Fruits and Vegetables

Currently, LK fruits and vegetables cultivation primarily focuses on controlling the K levels in nutrient solutions. However, the K content of fruits and vegetables is also affected by various factors, such as varieties [22], growing conditions [34], and exogenous substances [17]. Therefore, optimizing LK fruits and vegetables cultivation methods should consider these factors along with K regulation.

### 3.1. Utilizing High K Utilization Efficiency (KUE) Varieties

Varieties possessing high KUE genotypes offer significant advantages for LK fruits and vegetables cultivation. KUE refers to the dry matter production per unit of accumulated K, which is inversely related to the K content [35,36]. High KUE varieties typically exhibit a lower relative K content and greater tolerance to LK stress [37]. For example, among the lettuce varieties, crisphead demonstrated the lowest K content, 42.91% less than Romaine lettuce, because of its higher KUE [38]. Additionally, Yang [39] reported that K-efficient rice varieties (JNZ) yielded 1.59 times more grain than K-inefficient rice varieties (KQ47) under LK soil conditions.

Significant intraspecific variations in KUE have been documented across crops. Wu [40] examined K uptake and KUE among 56 barley varieties, revealing that the K dry matter production efficiency, K dry matter production index, and dry matter weight of the K-efficient genotype were 1.4–2.3, 2.1–3.9, and 1.7–2.1 times higher, respectively, than those of the K-inefficient genotype. The genotypic differences in KUE were primarily attributed to (1) the variances in K partitioning and redistribution at the cellular and whole plant levels, (2) the substitution ability of K by other ions [41], and (3) the proportion of resources allocated to economic products [36]. Table 1 illustrates the cultivars with high K efficiency and their phenotypes for certain crops. The K-efficient genotypes typically exhibit developed and dense roots [36,42,43,44,45,46,47], enhanced K uptake ability [40,44,45,48], efficient translocation and distribution of both K and carbohydrates [36,39,46], effective K recycling and reuse [37], greater Na substitution capacity [41], higher photosynthetic pigments [47], and a relative net photosynthetic rate under K deficiency [39]. Moreover, the K-efficient varieties demonstrate a higher grain-filling rate at maturity, increased grain weight per spike, and an elevated harvest index than K-inefficient varieties in rice [39] and wheat [49].

In LK fruits and vegetables cultivation, the standard practice involves providing a normal K supply in the early growth stages followed by K deficiency later on. Hence, selecting varieties capable of efficiently reutilizing the absorbed K is paramount. However, there is a dearth of research on this subject, making it a key area for future investigation of LK fruits and vegetables cultivation.

### 3.2. Optimize the Cultivation Temperatures

The temperature can significantly affect the plant growth and development, influencing the mineral absorption, distribution, transformation, and utilization efficiency [52,53]. Generally, the K uptake responds quadratically to temperature variations with the optimal temperatures for nutrient uptake and growth aligning closely [54,55,56,57]. Several studies have reported a positive correlation between K content and uptake. Increasing root zone temperature under lower air temperatures can enhance K uptake and elevate the shoot and root K content in crops such as tomatoes [58] and grapevines [59]. However, excessively high root-zone temperatures may hinder root development and reduce mineral absorption and transportation efficiency [60]. This can lead to decreased K content in plant leaves, as observed in various crops, such as salad rocket [34], cool-season grass [61], *Lactuca sativa* L. cv. *Panama* [62], and cucumber [63]. Nonetheless, the increase in biomass due to the appropriate temperatures may dilute the K in tissues, introducing uncertainty in the K content–temperature relationship. Studies have shown that the total K uptake in star fruit increases with increasing root temperature from 5 to 25 °C, with no significant difference in leaf K content [43], which is a finding corroborated by Boatwright [64]. Moreover, some studies have suggested that high temperatures exert a more pronounced negative impact on the KUE than on absorption efficiency, resulting in higher K content at elevated temperatures than under optimal conditions [54,56,65].

Although harsh ambient temperatures may decrease plant K content, the challenge of slowing growth rates and diminished marketability for commercial production cannot be ignored. The author suggests exploring alternative regulatory methods to reduce K content instead of inducing temperature stress.

### 3.3. Optimize Light Conditions

Light and K play pivotal roles in various biological processes including stomatal movement and crop quality. As key elements of light signaling, factors such as light intensity, quality, and photoperiod also govern the K uptake and utilization in plants [66]. Recently, researchers have investigated the influence of different light conditions on K absorption and concentration in plants (Table 2).

Previous studies have indicated that plant K uptake initially increases and then decreases with increasing light intensity with higher uptake occurring under moderate light conditions [54,67]. However, increased light intensity enhances the KUE, resulting in the decreased K content. This trend has been observed across various plant species, including basil [68], brassica microgreens [69], lettuce [54,70], herbaceous plants [71], *Tulbaghia violacea* L. [72], and *Erythrophleum fordii* Oliv. [73].

In addition to the light intensity, the K absorption and concentration are also influenced by the spectrum, although this effect may vary across different species or environmental conditions [74]. The multi-band composite spectra have been shown to mitigate damage caused by monochromatic light in *Coriandrum sativum* L. [75] and cucumber [76], resulting in a higher KUE and a lower K content compared to monochromatic light conditions. However, red light conditions led to the lowest K content in Chinese chive [77] and garlic seedlings [78]. In contrast, the K content of lettuce and Michaelmas daisy remained constant regardless of the presence of monochromatic or compound light in other experiments [79,80,81,82]. The impact of the red/blue (R:B) ratio on mineral absorption and accumulation has been extensively studied, as red and blue LEDs are commonly used for indoor plant cultivation [83]. The sweet basil presented an initial increase and subsequent decrease in K uptake and content as the R:B ratio varied from 0.5 to 4 with a peak uptake observed at 3 and the lowest at 0.5 [83]. Although Chen [84] also observed a similar pattern in lettuce, the correlation between the K content and the R:B ratio yielded inconsistent results across experiments. Wu [85] noted a decreasing trend in K content in lettuce as R:B ratios ranged from 100:0 to 60:40, whereas Liu [86] observed an initial increase followed by a decrease in K content as R:B ratios ranged from 3:1 to 1:3 with the highest content at 1:1 and the lowest at 3:1. However, studies by Chen [84] and Tian [87] did not find a correlation between the K content and changes in the R:B ratio. Under the identical R:B ratios, Kopsell [88] indicated that different wavelengths of blue light impacted mineral nutrients in kale (*Brassica oleracea* var. *acephala*) microgreens with lower blue LED wavelengths correlating with decreased K content. Green light has been associated with a reduced K content in lettuce [85] and flowering Chinese cabbage [89]. Plants grown under fluorescent lights (FLs) or high-pressure sodium lights (HPSs) with more green light bands exhibited lower K content than those grown under LED lights containing only red and blue light, supporting the hypothesis that green light reduces K content in plants [83,90,91]. Far-red (Fr) light also influences K absorption and utilization in certain plants. An increased proportion of far-red light promotes K absorption in lettuce [91] and the accumulation of K content per unit in tomato seedlings [92] while decreasing the K content in Chinese kale [93] and spinach [94]. The supplementation with trace amounts of UV-A has been shown to enhance basil growth and improve KUE, consequently reducing K content [95]. Although the UV-B radiation can reduce K content in some plants, its application is not suitable for LK vegetable production because of its predominantly injurious effects on plant growth [96].

Extending the light duration appropriately decreases the plant K content in some crops. Prolonged light exposure enhances K^+^ absorption by roots, its transport to the shoot, and the overall K accumulation per plant [97,98,99,100]. However, the relationship between K application efficiency and photoperiod varies among plants. Increasing the duration of light exposure generally enhances plant biomass accumulation while reducing K content. For example, Li and Liu [98] observed a reduction in cucumber shoot K content with three hours of nighttime light supplementation. Continuous lighting before harvest significantly decreases lettuce K content [86,99]. Han [101] reported that extending the photoperiod from 12 to 16 h increased the K content in leaves but decreased it in fruits. Furthermore, in experiments investigating light–dark rhythm effects on lettuce mineral content, researchers found lower K content with lower light–dark alternation frequencies [102].

In conclusion, utilizing specific light conditions to promote plant growth is effective in reducing K content, serving a dilution function [103]. This strategy is particularly advantageous for indoor cultivation, where artificial lighting is essential. Before the implementation, it is imperative to conduct experiments or refer to similar results from the literature to optimize light conditions for various plants to reduce K content.

**Table 2:** Differential light conditions affecting K uptake and concentration in various plant varieties.

Light Condition		Variation Trend	Plant Variety	K Uptake	K Concentration	References
Light intensity		Went from 7 to 15 (DLI, mol·m^−2^·d^−1^)	Basil		↓	[68]
		Went from 105 to 315 (PPFD, μmol·m^−2^·s^−1^)	Brassica microgreens		↓	[69]
		Went from 120 to 240 (PPFD, μmol·m^−2^·s^−1^)	Cucumber		↑	[104]
		Went from 100 to 600 (PPFD, μmol·m^−2^·s^−1^)	Lettuce	First↑ then ↓, highest on 500	↓	[54]
		Went from 300 to 450 (PPFD, μmol·m^−2^·s^−1^)	Lettuce		—	[87]
		Went from 150 to 450 (PPFD, μmol·m^−2^·s^−1^)	Lettuce		↓	[70]
		Went from 65 to 446 (PPFD, μmol·m^−2^·s^−1^)	New Guinea impatiens	Irregularity		[67]
		90% to 0% shading	*Dactylis glomerata*, *Festuca ovina*, *Trifolium subterraneum*, *Medicago lupulina*		↓	[71]
		15% to 100% full light	*Erythrophleum fordii* Oliv.		↓	[73]
		33% to 100% full sunlight	Tomato	↓		[105]
		40% to 0% shading	*Tulbaghia violacea* L.		↓	[72]
Light quality	Monochromatic light vs. compound light	R, B, G, 7R1B, 13R2B1Fr	*Coriandrum sativum* L.		The sequence from high to low: R, B, G, 13R2B1Fr, 7R1B	[75]
		R, B, 8R1B, W FL	Cucumber		The sequence from high to low: R, B, 8R1B, W	[76]
		R, B, 3R1B, 7R1B, W FL	Chinese chive		The sequence from high to low: B, 3R1B, 7R1B, W, R	[77]
		R, B, 3R1B, W FL	Garlic seedling		The sequence from high to low: B, 3R1B, W, R	[78]
		R, B, RB, UV-A, W FL	Lettuce (*Lactuca sativa* L. ‘Lollo Rosa’)		—	[81]
		R, B, RB, UV-A, W FL	Lettuce (*Lactuca sativa* L. ‘Chung Chi Ma’)		—	[82]
		R, B, 8R1B, W FL	Lettuce		—	[79]
		R, B, G, RB, YR, W FL	Michaelmas daisy		—	[80]
	R/B light ratio	R/B light ratio from 100:0 decreased to 0:100	Lettuce	First ↑ then ↓, highest on 70:30, lowest on 100:0	—	[84]
		R/B light ratio from 100:0 decreased to 60:40	Lettuce		↓	[85]
		R/B light ratio from 3:1 decreased to 1:3	Lettuce		First ↑ then ↓, highest on 1:1, lowest on 3:1	[86]
		R/B light ratio from 8:1 decreased to 1:1	Lettuce		—	[87]
		R/B light ratio from 4:1 decreased to 1:2	Sweet basil	First ↑ then ↓, highest on 3:1, lowest on 1:2	First ↑ then ↓, highest on 3:1, lowest on 1:2	[83]
		W LED, RB400, RB420, RB450, RB470	*Brassica oleracea* var. *acephala*		The sequence from high to low is: W, RB470, RB450, RB420, RB400	[88]
	G light	G light ratio rose	Lettuce		↓	[85]
		G/R light ratio rose	Flowering Chinese cabbage		↓	[89]
		W FL vs. RB	Broccoli		↓	[90]
		W FL vs. RB	Sweet basil		↓	[83]
		W HPS vs. RB	Lettuce		↓	[91]
	Far-red Light	Supplement far-red light	Chinese Kale		↓	[93]
		Supplement B, R, Fr LED to W FL	Lettuce		—	[106]
		Far-red/red light ratio rose	Lettuce	↑		[91]
		Supplement Fr to W LED light	Spinach		↓	[94]
		Supplement Fr	Tomato		↑	[92]
		Supplement Fr to R LED light	Tomato	—	—	[107]
	UV	Supplement UV-A	Basil		↓	[95]
		Supplement UV-B	Soybean seedlings		Root ↓, steam ↓, leaf ↑	[108]
		Supplement UV-B	Spring wheat		↑	[109]
		Supplement UV-B	Soybean		↓	[96]
		Supplement UV-B	Mono-maple seedlings		Root ↑, steam ↓, leaf ↑	[110]
		Supplement UV-B	Mung bean seedlings		Root ↑, steam ↓, leaf ↑	[111]
Photoperiod		Supplementary illumination duration from 0 h increased to 12 h	Cucumber	↑	Irregularity, lowest on 3 h	[98]
		12 h extends to 24 h	Lettuce	—	↓	[86]
		12 h extends to 16 h	Lettuce	↑		[100]
		Continuous lighting before harvest	Lettuce	↑	↓	[99]
		8/4 h (T12), 16/8 h (CK), 24/12 h (T36), 32/16 h (T48)	Lettuce	—	The sequence from high to low is: T12, T36, CK, T48	[102]
		Lengthen	Sweet Pepper	↑		[97]
		12 h extends to 16 h	Tomato		Leaf ↑, Fruit ↓	[101]

DLI: daily light integral; PPFD: photosynthetic photon flux density; LED: light-emitting diode; HPS: high-pressure sodium lamp; R, B, G, Y, Fr, UV-A, and UV-B represent red light LED, blue light LED, green light LED, yellow light LED, far-red light LED, ultraviolet-A radiation light LED, and ultraviolet-B radiation light LED; W FL and W HPS represent white fluorescent lamps and white high-pressure sodium lamps. ↓, ↑ and — represent that parameter was decreased, increased, and unaffected, respectively.

### 3.4. Optimizing Harvest Timing and Plant Parts Selections

The absorption and distribution of mineral elements in plants undergo dynamic changes during the various growth stages. K demonstrates high mobility, resulting in fluctuating levels in different plant parts throughout growth [112,113,114]. For instance, during onion growth stages, K absorption varied significantly: 3.32% at the seedling stage, 71.79% during vigorous growth, and 24.89% during bulb expansion. Distribution patterns also shift, with K primarily found in leaves at the seedling stage, in both bulbs and leaves during vigorous growth, and predominantly in bulbs during bulb expansion [115]. During cultivation, the K content in the roots, leaves, and bulbs initially rose and then decreased, stabilizing over time. After 15 weeks, the K contents per 100 g of FW were 188.9 mg in roots, 158.7 mg in leaves, and 88.3 mg in bulbs [11]. In hydroponic tomato cultivation, the K content in fruits initially increased, peaked at the third truss fruit, and then declined, with the fifth truss fruit exhibiting the lowest content [31]. Consequently, most of the K^+^ in leafy vegetables was concentrated in new leaves, allowing old leaves to be harvested and sold as LK vegetable commodities. In contrast, for bulb vegetables or fruits, suitable harvesting times and parts should be determined based on the distribution and variation in K content.

### 3.5. Application of Exogenous Substances

Certain exogenous substances can mitigate the effects of LK stress on LK fruits and vegetables production, thereby enhancing biomass and nutritional quality. Reported substances include proline [17], gibberellic acid-3 (GA3), jasmonic acid biosynthesis inhibitor (diethyldithiocarbamate; DIECA) [116], indole-3-acetic acid (IAA) [117], 1-naphthaleneacetic acid (NAA) [118], theanine [119], salicylic acid (SA) [120], and silicon [121]. For example, the application of exogenous proline notably increased the proline, soluble sugar, and ascorbic acid levels in LK lettuce. At concentrations of 1 and 1.5 mmol·L^−1^ proline, lettuce leaf superoxide dismutase (SOD) activity increased by 42.5% and 29.5%, respectively. Similarly, peroxidase dismutase (POD) activity increased by 67.0% and 39.3%, whereas shoot FW increased by 37.4% and 31.5%, respectively. Notably, the K content in proline-treated lettuce remained similar to that in untreated samples [17]. Compared to the control group, the supplementation with 0.1 μmol·L^−1^ GA3 increased shoot FW by 1.4 to 2-fold in both the LK-tolerant tomato cultivar, JZ34, and the LK-sensitive cultivar, JZ18. However, the K content in shoots decreased by 23.9% and 21.5% for JZ18 and JZ34, respectively. Similarly, application of 100 μmol·L^−1^ DIECA, significantly increased shoot FW by 1.3-fold under LK conditions while slightly reducing the K concentration in shoots [116]. Additionally, Liu [122] demonstrated that the exogenous hormone IAA improved the photosynthetic capacity and antioxidant enzyme activity in sweet potato under LK conditions, thereby reducing the ultrastructural damage in root tip cells. Thus, the appropriate addition of certain exogenous substances can alleviate K deficiency stress in LK fruits and vegetables production.

## 4. Shortcomings and Prospects of Research on LK Fruits and Vegetables

The LK fruits and vegetables lack standardized industry norms as functional foods for patients with CKD. Currently, there has been no widely accepted K content that defines the LK products. According to a summary of executive conclusions on hyperkalemia management in CKD published by Kidney Disease Improving Global Outcomes, foods with a potassium content of less than 200 mg·serving^−1^ are considered LK foods suitable for CKD patients [123]. A serving of fruit and vegetables is about 150 g and 200 g, respectively [18], so fruits and vegetables with a K content of less than 133 mg·100g^−1^ and 100 mg·100g^−1^, respectively, can be considered LK fruits and vegetables. This is in line with the standard for LK fruits and vegetables (less than 100 mg·100 g^−1^) currently sold in Japan, but further scientific verification is needed to ensure safety.

The cultivation methods for LK fruits and vegetables are relatively straightforward, often involving a K cut-off at later growth stages. However, this approach may lead to unstable K levels under various cultivation conditions. Although the cultivation primarily focuses on regulating the K content in the nutrient solution, the K levels are also influenced by genotype, growth environment, and other mineral nutrients. Several strategies can be implemented to optimize current cultivation methods. Firstly, K-efficient genotypes have been selected based on previous studies. Secondly, according to the type of fruits and vegetables, scientists can develop specific nutrient solution formula for low-potassium products. And a complete set of fertilization strategies should be developed according to the regulation strategies of irrigation frequency, nutrient solution EC, and pH. Thirdly, environmental controls (temperature and light) have been integrated to boost fruits and vegetables biomass, maximize the dilution effect to enhance KUE, and reduce plant K content. Fourthly, it can enhance the yield and quality by decreasing the K concentration through exogenous substance application, harvest timing, and plant parts selection. Finally, the comprehensive application of these methods can depend on the convenience of cultivation and the economy. In summary, the precise control of K content in fruits and vegetables requires in-depth study to develop a complete cultivation system and establish industry standards.

Currently, the concept of using LK fruits and vegetables for the nutritional treatment of CKD has remained unfamiliar to the public with limited clinical trial data available on their K control ability and safety for CKD patients. CKD nutritional treatment is multifaceted, necessitating the simultaneous consideration of protein, energy, Na, phosphorus, and K intake [18]. Research on LK fruits and vegetables has primarily focused on the K content and lacks comprehensive evaluation standards. Given the importance of fruits and vegetables as staple food items, it is vital to explore and establish accurate sales channels for LK products to meet population demand. Therefore, the research on LK fruits and vegetables should encompass multidisciplinary fields, such as agriculture, medicine, and business, to explore their clinical safety and nutritional treatment methods. Moreover, it is vital to explore potential application scenarios and suitable business models for LK fruits and vegetables to maximize their contribution to the nutritional treatment of CKD.

## Figures and Tables

**Table 1:** K-efficient genotype cultivar and K-inefficient genotype cultivar of some plant varieties, and characteristics of K-efficient genotype cultivar compared with K-inefficient genotype cultivar.

Plant Variety	K-Efficient Genotype	K-Inefficient Genotype	Phenotype of K-Efficient Genotype Cultivar	References
Barley	Sandrime	AC Westech	Higher K uptake, K dry matter production index, K dry matter production, efficiency, and dry matter weight	[40]
Cotton	103	122	Developed root systems, higher LK tolerance, better nutrition uptake capability, and stronger transport organs	[44,45]
Pear	*Pyrus ussuriensis*	*Pyrus betulifolia*	Higher LK tolerance, more efficiently recycles and reuses K	[37]
Potato	Huayu 5, Zhengshu 5	08CA0710, 09307-830, B20-7, Liangshu 2	Lower leaf and stem K content	[50]
Proso millet	Var 87, Var 189	Var 116	Developed and dense roots, higher photosynthetic pigments, and higher LK tolerance	[47]
Rice	HA-88, EJF, JNZ	KQ47, 81-280, TLHZ	Greater efficient translocation and distribution of both K and carbohydrate; higher relative net photosynthetic rate under LK supply; greater relative tillering rate during the tillering stage and a greater relative grain-filling rate during the late grain-filling stage	[39]
Sweet potato	Nan 88, Xushu 18, Shang 52-7, Zhe 6025	Zi 892, Meiguohong, Zi 1	Higher root weight and root: top ratio and harvest index (HI), and lower K concentration and accumulation	[43]
Sweet potato	Xu28, Wan5	Ji 22	Higher relative root weight per plant, lower K concentrations in the roots or whole plants at maturity, and better K translocation in the shoots and roots	[36,46]
Tea plant	1511	1601	Developed root systems and higher LK tolerance	[42]
Tobacco	Qinyan 96, Yuyan 6, Yunyan 87, Cuibi 1, Zhongyan 100	Eyan 1, RG17, Honghua Dajinyuan, G28, K326	Lower leaf and stem K content, root vigor, and K^+^ influxes	[51]
Tomato	576, 571	349, 203, 546	Higher Na substitution capacity	[41]
Watermelon	ZXG0516, ZXG1553, ZXG0620, YS	WFL, 8424, NBT, HJX1	Higher relative shoot dry weight (ratio between shoot growth at limited K and that at adequate K), higher K uptake ability, and lower K concentration	[48]
Wheat	Yunmei 5	94-18	Higher grain weight per spike and harvest index, lower stem K concentration at maturity	[49]

## Data Availability

All data are collected from open sources with detailed descriptions in the cited references.
